# Proton beam therapy for cervical lymph node metastasis in an octogenarian with melanoma of unknown primary: a case report

**DOI:** 10.1007/s13691-023-00597-8

**Published:** 2023-02-01

**Authors:** Masatoshi Nakamura, Kayoko Ohnishi, Fumihiko Uchida, Takashi Saito, Yuri Kitagawa, Ryota Matsuoka, Toru Yanagawa, Hideyuki Sakurai

**Affiliations:** 1 Department of Radiation Oncology, Faculty of Medicine, University of Tsukuba, 1-1-1 Tennodai, Tsukuba, Ibaraki 305-8577 Japan; 2 Department of Radiology, School of Medicine, International University of Health and Welfare, 4-3 Kozunomori, Narita, Chiba 286-8686 Japan; 3 Department of Oral and Maxillofacial Surgery, Faculty of Medicine, University of Tsukuba, 1-1-1 Tennodai, Tsukuba, Ibaraki 305-8575 Japan; 4 Department of Pathology, University of Tsukuba Hospital, 2-1-1 Amakubo, Tsukuba, Ibaraki 305-8576 Japan; 5 Department of Pathology, Faculty of Medicine, University of Tsukuba, 1-1-1 Tennodai, Tsukuba, Ibaraki 305-8577 Japan

**Keywords:** Melanoma of unknown primary, Proton beam therapy, Lymph node metastasis

## Abstract

An 80-year-old man with an approximately 3-cm mass in the right submandibular region presented to our institution. Magnetic resonance imaging revealed enlarged lymph nodes (LNs) in the right neck, and fluorine-18-2-deoxy-D-glucose (FDG) positron emission tomography (PET)/computed tomography (CT) indicated positive FDG accumulation in the right neck LNs only. Excisional biopsy was performed for suspected malignant lymphoma, and the biopsy revealed melanoma. Close examination of the skin, nasal cavity, oral pharyngeal and laryngeal cavities, and gastrointestinal tract were performed. No primary tumor was detected by these examinations, and the patient was diagnosed with cervical LN metastasis from melanoma of unknown primary of clinical stage T0N3bM0 stage IIIC. The patient refused cervical neck dissection because of his age and comorbidity of Alzheimer’s disease and instead opted for proton beam therapy (PBT) at a total dose of 69 Gy (relative biological effectiveness) in 23 fractions. He did not receive any systemic therapy. The enlarged LNs shrunk slowly, and FDG PET/CT at 1 year after PBT showed that the right submandibular LN had shrunk from 27 to 7 mm in length, and there was no significant FDG accumulation. At 6 years and 4 months after PBT, the patient is alive without any recurrence.

## Introduction

Melanoma of known primary (MKP) develops on the skin, eyes, and mucous membranes, while melanoma of unknown primary (MUP) is defined as melanoma discovered in subcutaneous tissue, lymph nodes (LNs), or visceral organs without a primary site [1]. MUP is rare, accounting for approximately 3% of all melanomas, and 40–60% of MUP cases involve the LNs, including the axilla (50%) and cervical (30%) LNs [2]. MUP presents at a median age of 40–50 years and exhibits a male preponderance (male:female ratio ~ 2:1) [2].

The standard definitive treatment for melanoma of the head and neck is surgery, radiotherapy including particle therapy, or surgery followed by radiotherapy; the prognosis is poor regardless of the treatment. On the other hand, the standard definitive treatment for MUP is surgery, and MUP reportedly has a better prognosis than that of MKP, suggesting that these two diseases potentially have different characteristics and treatment outcomes [3]. Because MUP is rare, outcomes after treatments other than surgery have hardly been reported, including in case reports. We report an octogenarian patient with cervical LN metastasis from MUP who refused surgery due to his advanced age and comorbidity of Alzheimer’s disease and who was successfully treated with proton beam therapy (PBT).

## Case report

**Fig. 1:**
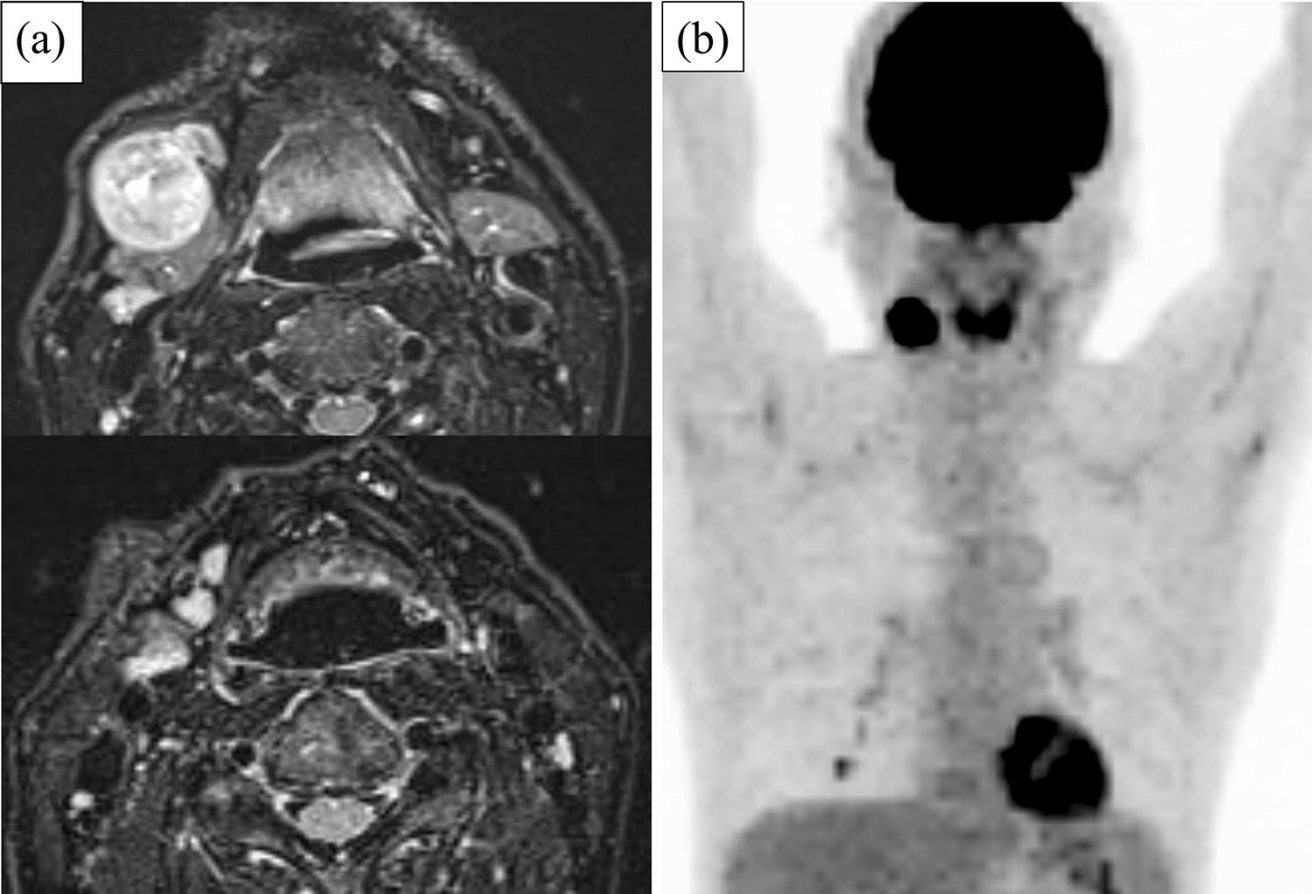
Magnetic resonance imaging and maximum intensity projection positron emission tomography (PET) images at initial diagnosis. **a** Short TI inversion recovery images showed a lymph node (LN) of approximately 2.7 cm in maximum diameter in the right submandibular region and LNs of approximately 1.0 cm in the right upper jugular region. **b** PET showed significant positive fluorine-18-2-deoxy-D-glucose accumulation in the LNs in the right submandibular and upper jugular regions

**Fig. 2:**
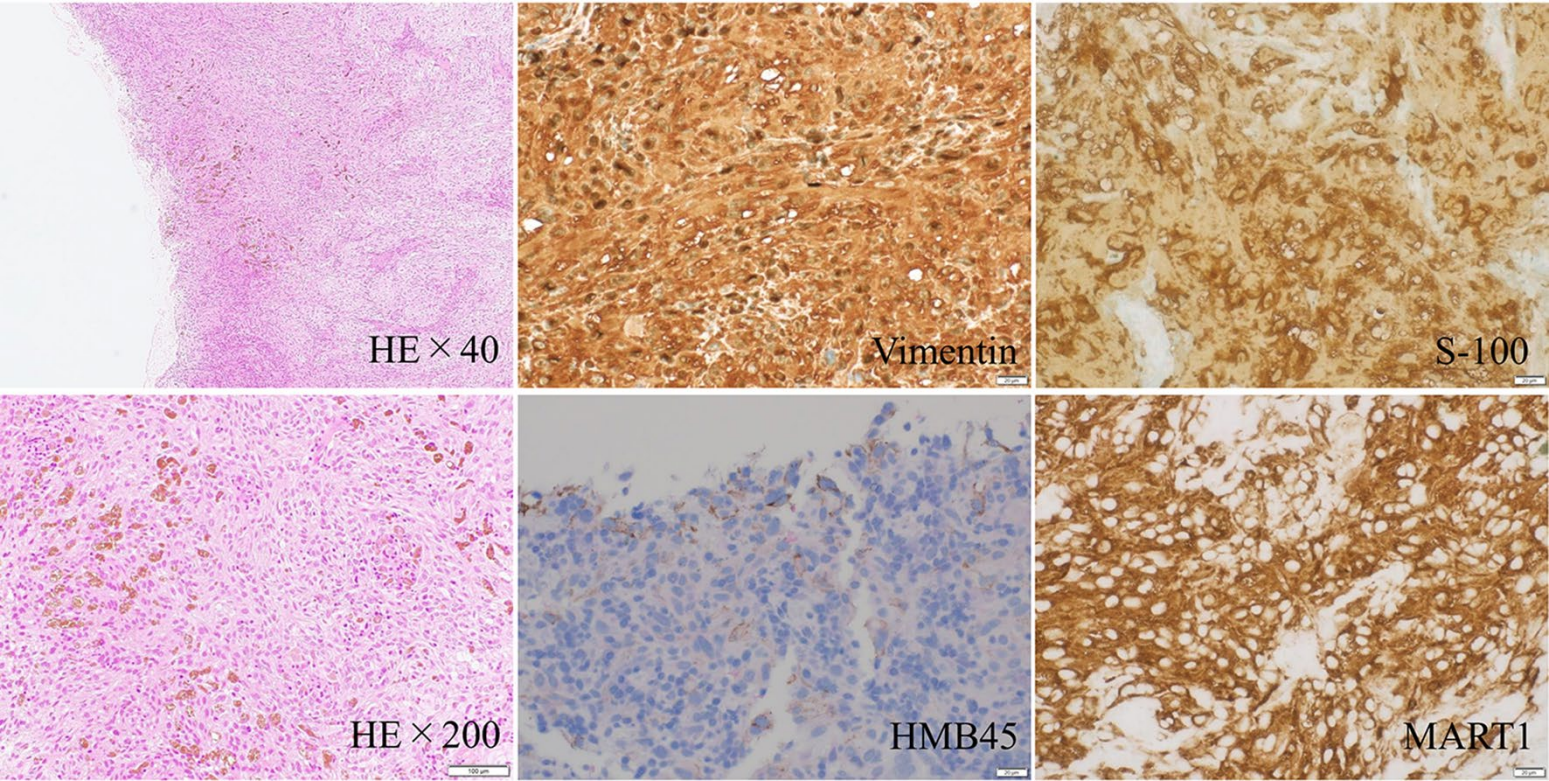
Microscopic images of hematoxylin–eosin and immunohistochemical staining

An 80-year-old man with a 3-cm tumor in the right submandibular region presented to our institution. At the first visit, his Eastern Cooperative Oncology Group performance status was 0. Magnetic resonance imaging of the neck revealed a LN of 2.7 cm in maximum diameter in the right submandibular region, and six LNs measured approximately 1.0 cm in the right upper jugular region (Fig. 1a). These LNs showed low signal intensity on T1-weighted images, high signal intensity on diffusion-weighted images, and low apparent diffusion coefficient values. Fluorine-18-2-deoxy-D-glucose (FDG) positron emission tomography (PET)/computed tomography (CT) indicated significant positive FDG accumulation in the right neck LNs only; consequently, malignant lymphoma was suspected. A maximum intensity projection PET image is shown in Fig. 1b. Excisional biopsy was performed, and the tumor was grayish-white macroscopically. Histological evaluation revealed a sheet-like growth of strongly atypical tumor cells with extensive necrosis and melanin granules in tissues stained with hematoxylin–eosin (Fig. 2). Immunohistochemistry showed positive staining for vimentin, HMB45, S-100, and MART1. Based on these findings, the tumor was diagnosed as malignant melanoma. Programmed death ligand 1 and BRAF mutations were not assessed at the time of diagnosis.

**Fig. 3:**
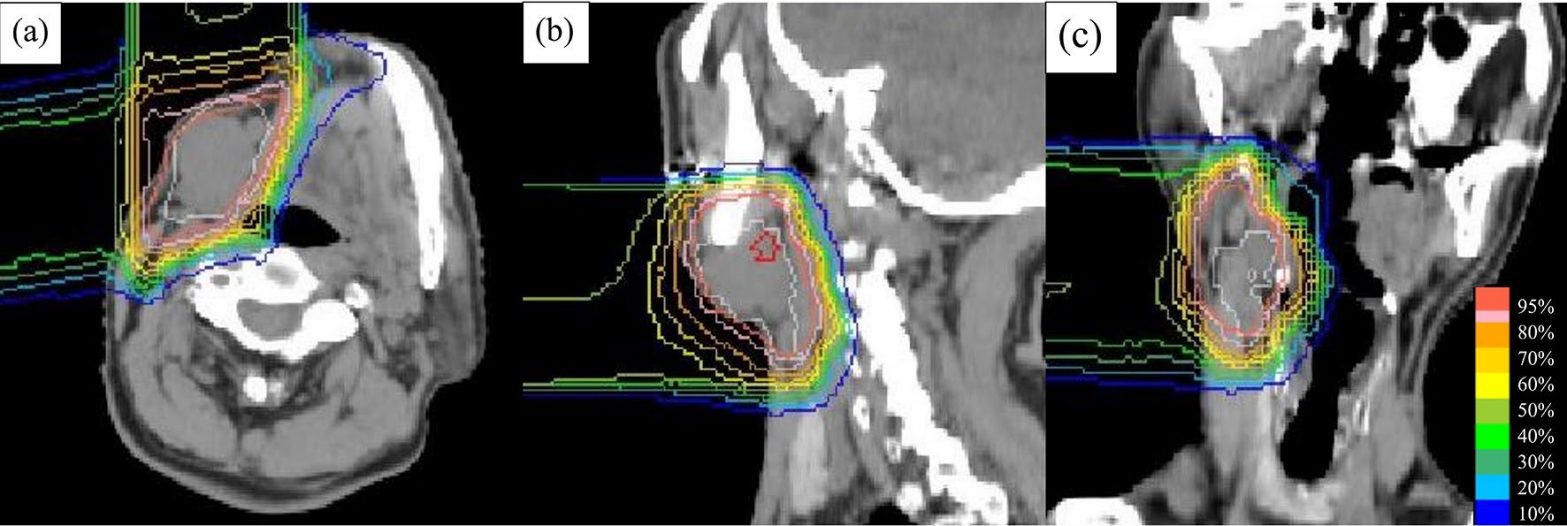
Dose distribution of proton beam therapy. The irradiated fields encompassed the clinically positive LNs and surrounding enlarged LNs in the level Ib, II, and III areas of the right neck. Isodose curves on axial (**a**), sagittal (**b**), and coronal (**c**) images

The search for the primary site of the melanoma was performed by a dermatologist, gastroenterologist, and otorhinolaryngologist, but no primary site was obvious in the external or middle ear, nasal cavity, oral pharyngeal and laryngeal cavities, esophagus, stomach, duodenum, or cutaneous region. Thus, the patient was diagnosed with cervical LN metastasis from MUP of clinical stage T0N3bM0 stage IIIC according to the 8th edition of the American Joint Committee on Cancer staging manual. Although neck dissection was recommended as the primary treatment option, the patient and his family refused surgery due to his advanced age and comorbidity of Alzheimer’s disease. Systemic therapies, such as programmed death 1 antibody and BRAF/MEK inhibitors, are a treatment option for inoperable patients with stage IIIC melanoma; however, these drugs were not covered by national health insurance yet, and the patient opted for PBT instead of chemotherapy using dacarbazine. Passive-scattering PBT was delivered using anterior and right lateral beams. A dose of 3 Gy (relative biological effectiveness [RBE]) per fraction, 5 times per week, for a total dose of 69 Gy (RBE), was prescribed. The radiation field encompassed the clinically positive LNs and surrounding enlarged LNs at level Ib, II, and III in the right neck (Fig. 3). After delivering a dose of 60 Gy (RBE), the treatment plan was slightly modified to reduce the dose to the pharynx. The maximum dose, as the equivalent dose in 2 Gy fractions (EQD2), to the pharynx was 71.7 Gy (RBE). PBT was completed without interruption in an outpatient setting, and the only acute adverse events experienced were grade 3 dermatitis and grade 2 mucositis according to the Common Terminology Criteria for Adverse Events, version 5.

**Fig. 4:**
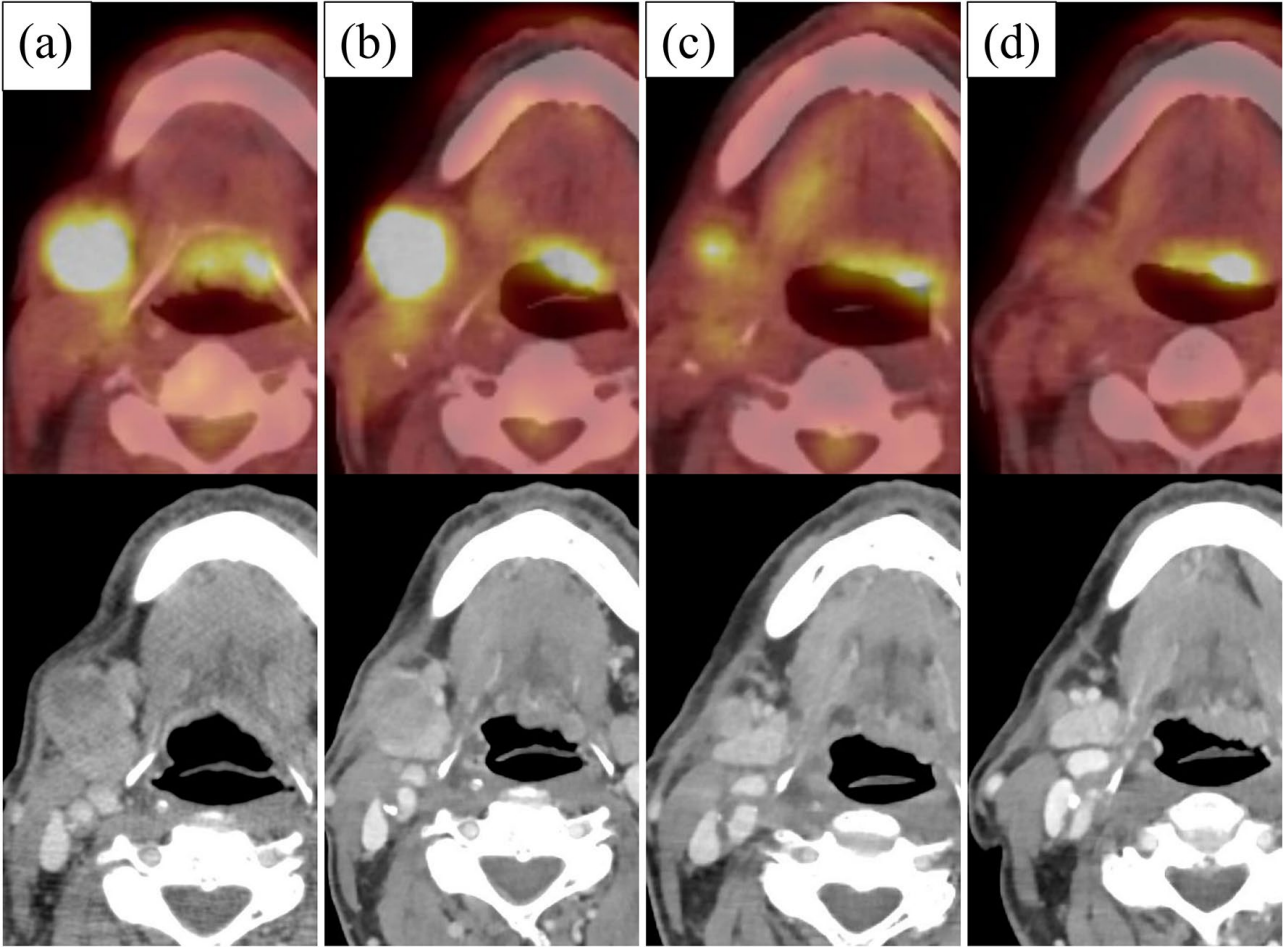
Contrast-enhanced computed tomography and positron emission tomography findings after definitive proton beam therapy. **a** At pretreatment, the tumor had a diameter of 2.7 cm and fluorine-18-2-deoxy-D-glucose (FDG) accumulation. **b** At 3 months after proton beam therapy initiation, the tumor diameter was reduced from 2.7 to 2.5 cm, but the FDG accumulation was not reduced. **c** At 6 months, the tumor diameter had shrunk further to 1.3 cm, and the FDG accumulation had diminished. The lymph nodes in the right neck had also shrunk. **d** At 12 months, the tumor diameter was further reduced to 0.7 cm, and the FDG accumulation was no longer apparent

Serial FDG PET/CT images before and after PBT are shown in Fig. 4. At 3 months after PBT initiation, FDG PET/CT indicated that the tumor diameter had shrunk from 2.7 to 2.5 cm, but the FDG accumulation had not decreased. However, at 6 months, the tumor diameter had shrunk further to 1.3 cm, and FDG accumulation had diminished; the LNs in the cervical area had also shrunk. At 12 months, the tumor diameter was further reduced to 0.7 cm, and FDG accumulation was no longer apparent. Thereafter, PET/CT or enhanced CT was performed every 6 months, and no local recurrence or distant metastasis was observed at 6 years and 4 months after PBT. No late adverse event of grade 2 or higher was seen.

## Discussion

The mechanism and pathogenesis of MUP are considered to be largely related to the spontaneous regression of melanoma from a known primary site [4]. The spontaneous regression of melanoma likely involves both cell-mediated and humoral immune mechanisms, and melanoma-specific antibodies are highly prevalent in the serum of MUP patients [5]. It has been reported that an increased number of tumor-infiltrating lymphocytes is a favorable prognostic factor for MUP [6]. Other factors that may induce the development of MUP include ectopic melanocytes that reach the LNs via the dermis and lymphatic vessels [7].

Dasgupta et al. proposed the following exclusion criteria for the diagnosis of MUP to avoid missing the primary site: (i) evidence of previous orbital exenteration or enucleation; (ii) evidence of a previous skin excision, electrodessication, cauterization, or other surgical manipulation of a mole, freckle, birthmark, paronychia, or skin blemish; (iii) evidence of metastatic melanoma in a draining lymph node and a scar in the skin area supplying the lymph node basin; and (iv) lack of a thorough physical examination, including ophthalmologic, anal, and genital exams [1]. However, fewer than 20% of reported MUP cases meet these exclusion criteria, and there is still insufficient consensus on the detection of the primary site [2]. In this case, although examination of the perineal region and eyes was insufficient, we considered the diagnosis of MUP to be adequate because the patient has been followed up with a physical examination, PET, and enhanced CT for more than 6 years without identification of a primary tumor since PBT.

Surgical management is a standard treatment for regional metastasis from MUP. In a matched-pair analysis of stage III patients who were managed with regional lymphadenectomy for nodal metastasis from MUP or MKP, the 5-year overall survival (OS) rate was significantly higher for MUP patients than MKP patients (58% vs. 40% *p* = 0.0006) [8]. Moreover, local control improved the 5-year OS rate in patients with lymph node metastasis from MUP (54% in the radical neck dissection group vs. 26% in the no dissection group), and surgery not only reduced the tumor volume but also stimulated anti-tumor immunity by downregulating immunosuppression toward the tumor, suggesting the importance of definitive local treatment for lymph node metastasis from MUP [1, 9]. In our case, radical neck dissection was not performed, but local therapy with PBT achieved good local control, which probably increased the survival of the patient.

Melanoma is radioresistant. Overgaard et al. analyzed 204 melanomas treated with fractionated radiotherapy assuming a low α/β value of 2.5 based on the linear–quadratic model, which describes the relationship between cell survival and the delivered dose of fractionated radiotherapy; this suggests that hypofractionated radiotherapy is better than conventional fractionated radiotherapy at improving local control [10]. That study also showed that a high total biologically effective dose (BED), which was calculated based on the linear–quadratic model with an α/β value set to 2.5, was associated with better tumor response, and the BED for a 50% complete response was estimated to be 125 Gy. In fact, in a multi-institutional retrospective study of radiotherapy for mucosal melanoma of the head and neck in Japan, Wada et al. reported that a hypofractionated radiotherapy dose ≥ 3 Gy per fraction and a high BED ≥ 118 Gy resulted in significantly better local control and OS rates [11]. In the present case, hypofractionation of 3 Gy (RBE) per fraction and a high total dose of 69 Gy (RBE), which was a BED of 151.8 Gy (RBE), resulted in good local control. Meanwhile, proton beams reduced the irradiated doses and volumes in the pharynx, and the treatment was well tolerated despite the patient’s age, with no acute or late mucositis of grade 3 or greater observed.

Today, with the widespread use of various high-precision radiotherapy techniques, an increasing number of patients are receiving definitive radiotherapy via stereotactic body radiotherapy or intensity-modulated radiotherapy using X-rays [12, 13]; however, particle therapy is preferable because it can deliver high doses while reducing the doses to surrounding organs [14]. In Japan, particle therapy for melanoma of the head and neck is covered by national health insurance, and it could be an effective treatment option for melanoma in patients who are not candidates for surgery or who refuse surgery.

In conclusion, we report a case of long-term survival after definitive PBT of cervical lymph node metastasis from MUP. Local control of MUP affects the survival rate, and high-dose hypofractionated radiotherapy could be an option for inoperable patients.

## Data Availability

The datasets used and/or analyzed during the current study are available from the corresponding author on reasonable request.
